# Changing our way of working for a greater integration of mental health patients: The evolution of the Zamora’s Assertive Community Treatment over the last 10 years

**DOI:** 10.1192/j.eurpsy.2024.475

**Published:** 2024-08-27

**Authors:** L. Vicente Rodriguez, L. Giménez Robert, L. Carrascal Laso, V. Saez Enguidanos, R. S. Gamonal Limcaoco, M. A. Franco Martin

**Affiliations:** ^1^Psychiatrist Department, Complejo Asistencial Zamora, Zamora, Spain

## Abstract

**Introduction:**

Since its beginning in the 1970s in Wisconsin, Assertive Community Treatment (ACT), has been adopted by numerous hospitals worldwide. It improves outcomes for people who are most at-risk of psychiatric hospitalization. The main goal is to provide a global attention with a focus on promoting maximum autonomy and facilitating integration into society. In 2012, the Health Care Complex of Zamora, Spain, adopted this pioneering approach to Mental Health. The main efforts were focused on creating a community network for individuals with severe mental disorders. It embraced a biopsychosocial model of intervention aimed at facilitating patient recovery, giving them tools to create a new life project based on their own autonomy.

**Objectives:**

The primary objective of this study was to assess the progress of the Assertive Community Treatment (ACT) since its introduction at the Health Care Complex of Zamora, with a specific focus on analyzing the number of hospitalizations as the dependent variable.

**Methods:**

A quantitative analysis about psychiatry number of hospitalizations was conducted using the database of the Zamora’s Psychiatry Hospitalization Unit. SPSS Statistics for Windows was used to calculate statistical values related to number of hospitalization. The dataset covers de period from 2010 to 2017.

**Results:**

The implementation of ACT has resulted in a significant reduction in hospitalizations reaching up to 75% in the Psychiatry Service of Zamora. It has been revealed a decrease from 17107 hospitalizations registered in 2011 to a total reduction to 4869 stances in 2013. A consistent trend in the reduction of hospitalizations has been observed (figure 1).A restructuration of the Hospitalization Unit was performed in order to implement de community model and reduce hospitalizations. Removal of more than 50% of the beds was developed.Besides, there has been implemented a community subunit with the objective of regaining their autonomy after a psychiatric exacerbation.

**Image:**

**Figure:**
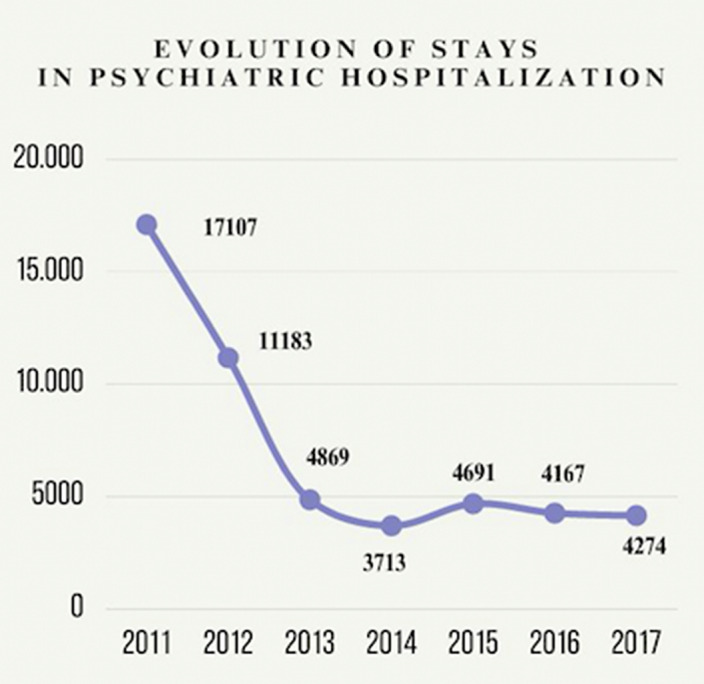

**Conclusions:**

Getting hospitalized in a Psychiatry Unit can have many different socio-laboral consequences. The ACT model has demonstrated a significative reduction in hospitalizations and it has evolved into a support network dedicated the integration of individuals that are usually left behind by society. Moreover, it presents itself as a positive cost-benefit intervention.ACT allows us to envision a future with fewer hospitalization and greater integration of mental health patients into modern society.

It is important to emphasize that the city of Zamora possesses unique characteristics that have facilitated the adaptation of this model. Not only are the rental prices for housing usually affordable, but the city’s small size, which easy walking, allows for easy access to Community Mental Health resources and services.

**Disclosure of Interest:**

None Declared

